# Effectiveness of Couple-Based HIV Counseling and Testing for Women Substance Users and Their Primary Male Partners: A Randomized Trial

**DOI:** 10.1155/2013/286207

**Published:** 2013-03-12

**Authors:** James M. McMahon, Stephanie Tortu, Enrique R. Pouget, Leilani Torres, William Rodriguez, Rahul Hamid

**Affiliations:** ^1^School of Nursing, University of Rochester Medical Center, 601 Elmwood Avenue, BOX SON, Rochester, NY 14642, USA; ^2^School of Public Health, Health Sciences Center, Louisiana State University, 2020 Gravier Street, 3rd Floor, New Orleans, LA 70112, USA; ^3^National Development and Research Institutes, 71 West 23rd Street, 8th Floor, New York, NY 10010, USA

## Abstract

A randomized trial was conducted to test the effectiveness of couple-based HIV counseling and testing (CB-HIV-CT) and women-only relationship-focused HIV counseling and testing (WRF-HIV-CT) in reducing HIV risk compared to the National Institute on Drug Abuse HIV-CT standard intervention. Substance using HIV-negative women and their primary heterosexual partner (*N* = 330 couples) were randomized to 1 of the 3 interventions. Follow-up assessments measuring HIV risk behaviors and other relevant variables were conducted at 3- and 9-months postintervention. Repeated measures generalized linear mixed model analysis was used to assess treatment effects. A significant reduction in HIV risk was observed over the 9-month assessment in the CB-HIV-CT group compared to that of the control group (*b* = −0.51, *t*[527] = −3.20, *P* = 0.002) and compared to that of the WRF-HIV-CT group (*b* = −0.34, *t*[527] = −2.07, *P* = 0.04), but no significant difference was observed between WRF-HIV-CT and controls (*b* = −0.17, *t*[527] = −1.09, *P* = 0.28). A brief couple-based HIV counseling and testing intervention designed to address both drug-related and sexual risk behaviors among substance using women and their primary male partners was shown to be more effective at reducing overall HIV risk compared to a standard HIV-CT intervention in an urban setting.

## 1. Introduction

Over the past two decades women have increasingly shouldered the burden of the HIV/AIDS pandemic. From 1997 to 2007 the female proportion of people living with HIV/AIDS globally rose from 41% to 50% [1, 2]. In the United States, the proportion of women representing new AIDS cases has more than tripled since 1985, from 8% to 27% [3]. This trend is most evident among African American and Latina women, who make up 24% of the female population in the USA but account for 82% of women living with HIV/AIDS [3]. AIDS remains the leading cause of death for African American women aged 25 to 34, and it is the second and third leading cause of death, respectively, for African American and Latina women between 35 to 44 years of age. About 80% of all incident HIV infections among minority women are sexually acquired, primarily from minority men [3]. In many low-income urban and rural communities across the USA the HIV/AIDS epidemic among African American and Latina women has reached alarming levels eliciting warnings of a “state of emergency” [4] and “an insidious epidemic… that demands immediate attention” [5].

Recent evidence has further shown that most women who become HIV infected acquire the virus from a husband or other primary male partner [1, 6–17]. In our work with drug-using minority women in East Harlem, New York City, the relative risk of incident HIV infection attributable to a primary male partner was 2.4 times the risk from nonprimary partners (i.e., casual and commercial sex partners) [14]. Similarly, Kalichman et al. [16] estimated that HIV transmission rates were nearly double for women with a primary male partner compared to those women with nonprimary partners. In a study conducted by Wilson et al. [15], epidemiological modeling revealed that Latina women's risk for acquiring HIV from a primary partner was more than 6 times greater than from a nonprimary partner. And Finer et al. [11], using data from several large national surveys, estimated HIV risk exposure for women aged 15 to 44 in the USA and concluded that nearly 1 million more women were at risk from a primary male partner than from nonprimary partners. These and other studies have led HIV prevention researchers to conclude that: “the majority of U.S. women with HIV have been infected via heterosexual intercourse in an intimate relationship” [17].

Another important risk factor for HIV infection among African American and Latina women involves the use of illicit drugs. Numerous studies dating back to mid 1990s have shown that drug-using women and men and their sexual partners are at heightened risk for HIV [18–20]. The 1996 National Household Survey on Drug Abuse (NHSDA), which surveyed 12,381 USA adults ages 18 to 59, found that “persons who were at risk through drug behavior were much more likely than others to be at risk through sexual behavior” [19]. Local HIV/AIDS epidemics in high-risk urban areas such as Harlem and the South Bronx in New York City tend to be concentrated among drug users and their sexual partners. The NHSDA study concluded that “the high rate of sexual risk behavior on the part of drug users suggests that increasing condom use for this group should be a priority goal for programs, especially condom use with main partners” [19]. Drug-involved women are especially vulnerable to HIV due to the large number of infected males in economically disadvantaged urban neighborhoods [21]. Additionally, drug-using women in primary relationships often underestimate their risk from a primary male partner [22]. In our research with drug-using heterosexual couples in high HIV prevalent neighborhoods in New York City, we found that nearly one-third of couples who self-reported HIV-negative status were actually HIV serodiscordant. And among male-positive/female-negative couples, more than two-thirds reported unprotected vaginal or anal sex in the last 30 days. Moreover, even women who were aware of their male partner's HIV status or high-risk behaviors continued to have unprotected sex: 56% of women who had unprotected sex with an infected partner had knowledge of his HIV-positive status [14]—so-called “informed exposure.” Thus, due to multiple biological, epidemiological, and psychosocial factors, drug involved African American and Latina women in primary relationships with men constitute one of the most vulnerable risk groups for HIV in the USA and merit high priority for HIV prevention research and interventions.

Given these trends, HIV prevention efforts over the last 15 years have focused primarily on women, with relatively little knowledge gained on the role that men play in drug and sexual risk and protective behaviors. There is a growing consensus, however, that the next generation of prevention programs need to include a greater emphasis on contextual, relationship, and male gender perspectives. In the context of HIV risk, men's influence on sexual decision making is particularly salient; de Zoysa et al. [9] referred to men as the “gatekeepers to sexual and reproductive behavior change.” Not surprisingly, the female-centered approach has not had the desired impact of stemming HIV epidemics among at-risk minority women and girls. There have been successes, to be sure, but even these have been limited by an emphasis on multisession women-only programs that do not fully capitalize on the dyadic nature of injection and sexual risk behavior or the difficulty that drug-using women (especially those out-of-treatment) face in attending multiple intervention sessions.

To address these limitations, we designed a brief couple-based HIV risk reduction intervention modeled on the HIV counseling and testing (HIV-CT) delivery modality. An individual woman-only *relationship-focused* HIV-CT was also developed. In this paper we report the results from the Harlem River Couples Project, a randomized clinical trial (NCT00325585) designed to evaluate HIV risk reduction effectiveness of the two experimental interventions compared to the standard-of-care National Institute on Drug Abuse (NIDA) individual HIV counseling and testing intervention. We hypothesize that actively engaging both the female and male partner in joint couple-based counseling and testing (CB-HIV-CT) will yield better risk reduction outcomes than either the woman-only relationship-focused model (WRF-HIV-CT) or the standard NIDA HIV-CT and that the WRF-HIV-CT intervention will result in greater risk reduction than the NIDA control. 

## 2. Methods

### 2.1. Study Design and Participants

Reporting of study design and results are consistent with the CONSORT statement [23] and the Centers for Disease Control and Prevention (CDC) Synthesis Research project guidelines [24]. A total of 330 sexually active heterosexual couples (660 individuals) were recruited from Central and East Harlem and South Bronx in New York City from March 2005 to September 2007. After enrolment and completion of baseline assessment, each couple was randomly assigned to one of three HIV counseling and testing interventions: (a) couple-based HIV-CT, (b) woman-only relationship-focused HIV-CT, or (c) NIDA standard HIV-CT (control). The CB-HIV-CT was administered to both female and male members of couples jointly, whereas only women participated in the WRF-HIV-CT and NIDA HIV-CT interventions. Followup assessments were conducted at 3- and 9-month postintervention. Both female and male members of each couple were invited to attend followup assessments regardless of intervention allocation. For couples randomized to one of the woman-only individual interventions, male partners were offered standard NIDA HIV-CT at terminal followup. Study activities were performed at a field office located in the South Bronx. 

Eligibility criteria were based almost exclusively on female characteristics, including (a) 18 years of age or older, (b) self-reported use of crack/cocaine or heroin (injected or noninjected) in prior 30 days, (c) current male sex partner identified as primary partner for at least six months, (d) had unprotected vaginal or anal sex with primary partner in prior 30 days, (e) able to enlist male partner into the study, (f) would not feel threatened participating in the study with primary partner, (g) must not have participated in HIV/AIDS related study or attended HIV counseling and testing session in prior 6 months, (h) self-reported HIV seronegative, and (i) fluent in English or Spanish. Male partners of eligible women must have been 18 years of age or older to participate. We defined primary partner as “a husband, common-law husband, or steady boyfriend of at least 6 months,” which had good face validity in the context of the study population [25].

### 2.2. Recruitment and Randomization

Procedures for the recruitment and enrollment of couples into the trial have been described in detail by McMahon et al. [26]. In order to protect women against potential partner conflict and violence, couples were recruited through the female partner to give women advance opportunity to learn about the study and decline involvement if they felt threatened or uncomfortable participating [26]. Women who were eligible and willing to participate were asked to enlist their male partner into the study, following a set protocol. An adaptive sampling and recruitment strategy was employed that combined respondent-driven sampling (RDS) with targeted sampling of female “seed” participants from street locations and venues identified through ethnographic mapping [27–29]. A total of 432 women were screened for eligibility; 90 (21%) did not meet the eligibility criteria. The remaining 342 couples visited the South Bronx field office for baseline enrollment. A screening tool was administered to validate the primary partnership status of couples; 12 couples failed this screening [26]. Each of the remaining 330 women and their 330 primary male partners provided written informed consent and were enrolled in the trial. Once enrolled, each member of the couple was escorted to a separate private office and administered a structured quantitative questionnaire by gender-matched bilingual interviewers. A combination of computer-assisted personal interview (CAPI) and, for sensitive items, audio computer-assisted self-interview (ACASI) was used to administer the questionnaires [30]. The average duration of interviews was 54 minutes (Std. Dev. 14 minutes). 

After completion of concurrent individual female and male baseline interviews, each couple was randomly assigned to one of the three treatment arms. Randomization was performed at the field office by the Project Director and one staff witness using a true random number generator [31]. Allocation results were CB-HIV-CT, 110 couples (33.3%); WRF-HIV-CT, 104 couples (31.5%); and NIDA HIV-CT standard-of-care control, 116 couples (35.2%). Three-month and 9-month followup assessments were conducted either with women alone or simultaneously with both members of the couple (men were not permitted to attend followup unaccompanied by their female partner). All study protocols were approved by an Institutional Review Board.

### 2.3. Interventions

#### 2.3.1. NIDA HIV-CT (Standard-of-Care Control)

The standard-of-care control condition was the National Institute on Drug Abuse (NIDA) Community-Based Outreach Model, which is a manualized HIV counseling and testing protocol for substance users [32]. The intervention is administered to individuals and consists of two sessions: (1) pretest counseling and voluntary HIV and hepatitis B and C testing and (2) posttest results and risk reduction reinforcement. The counseling component of Session I provides clients with information about risky behaviors, as well as concrete strategies and behavioral skills for risk reduction. Interventionists use a series of 24 cue cards to guide the sessions based on the individual's risk profile. Topics covered include basic information about HIV, HBV and HCV; injection-related risks and prevention strategies; sex-related risks and prevention strategies; cessation of drug use and benefits of drug treatment; and information about HIV antibody testing. Materials supporting risk reduction are distributed, and written referrals for drug treatment and other social and medical services are provided. Procedures for voluntary HIV and hepatitis B and C antibody testing are described and testing is offered. Session II is administered several weeks after testing and is designed to provide individuals with the provision and meaning of test results, along with a risk-reduction booster session. Different content is provided to clients who test seronegative compared to those who test seropositive. HIV treatment options and partner notification are discussed with the latter. Interventionists may be certified HIV/AIDS educators or individuals with prior experience as outreach workers or HIV counselors. Moderate training and supervision are required depending on the experience and skills of the interventionist. 

#### 2.3.2. Couple-Based HIV-CT (CB-HIV-CT)

The CB-HIV-CT follows the standard model of voluntary HIV counseling and testing divided into pre and posttest sessions. After a brief introduction to the intervention to begin the pretest and testing session, the counselor administers a short dyadic risk assessment that permits the intervention to be tailored to each couple's risk profile, stage of behavior change for various risk behaviors, and self-reported dyadic HIV serostatus (i.e., concordant negative/discordant). Basic information about HIV and other sexually transmitted infections, including hepatitis B and C, is provided to all couples. Thereafter, each couple is administered a series of risk reduction minisessions based on their risk profile. These minisessions address (1) regular recurrent HIV/STD testing, (2) condom use to prevent HIV/STDs, (3) anal sex and HIV/STD risk, (4) safe injection practices, (5) safe noninjection drug use practices, (6) sexual risk reduction when trying to conceive, (7) adherence to HIV treatment (for serodiscordant couples), (8) hepatitis B and C treatment, and (9) substance use and treatment. Not all couples receive all minisessions. For example, if neither member of the couple is an injection drug user then the minisession on safe injection practices would be excluded. The content of selected minisessions is further customized to accommodate couples' stage of behavior change. For example, the intervention content for at-risk couples who are not using condoms (preaction stage) focuses on behavior change (e.g., addressing barriers to change) whereas the intervention content for couples using condoms intermittently or consistently (postaction stage) focuses on increasing or maintaining existing condom use (e.g., positive reinforcement).

After completion of the minisessions, interactive exercises are conducted that address negative norms related to risk behavior as well as couples' communication skills that may inhibit enactment of preventive behavior. Throughout the session, the counselor maintains an action plan of activities each couple has agreed to perform after completion of the intervention, for example, to desist or limit unprotected anal sex or engage in safe injection practices. Some action plan elements involve active referrals by the counselor, such as enrolling in a drug treatment program. The last component of the couples joint counseling session is to provide pretest information regarding HIV and hepatitis B and C antibody testing. Biological sample collection for testing is performed with each member of the couple individually. During these individual testing sessions the counselor addresses sex and drug-related risk the client might be engaged in outside of their primary relationship. Finally, the counselor or phlebotomist performs the biological sample collection procedures for testing. Consistent with the HIV-CT model, the couple is asked to return for their test results, and the counselor performs couple-based posttest counseling in which test results are provided and the couple's action plan is reviewed for compliance. 

#### 2.3.3. Woman-Only Relationship-Focused HIV-CT

To assess whether any observed intervention effects are due to participant modality (joint couple's counseling versus individual counseling) and not simply to intervention content (relationship-focused versus individual-focused), the structure and content of the woman-only relationship focused HIV-CT intervention were matched as closely as possible with that of the couple-based HIV-CT. Therefore, the initial risk assessment and minisessions focus on women's risk with a primary male partner. The same interactive exercises conducted as part of the CB-HIV-CT were adapted to be performed individually by women rather than jointly by the couple. Individual action plans are developed. Women's sex and drug-related risk with secondary partners are addressed just prior to HIV and hepatitis B and C testing. Posttest counseling is also conducted individually with women, the content of which follows closely that of the CB-HIV-CT, with an added discussion regarding partner notification in the case of a positive test result.

Development of the couple-based and women-only HIV-CT experimental interventions was informed by an integrated theory of HIV risk that incorporated elements of Social-Cognitive Theory [33], Information-Motivation-Behavior Skills model [34], Stages-of-Change model [35], and the Theory of Gender and Power [36, 37]. 

### 2.4. Interventionists and Intervention Fidelity

One male bilingual (English and Spanish) interventionist performed 95% of the 330 HIV counseling and testing interventions administered across the three conditions. The remainder was performed by one female bilingual back-up interventionist. The principal male interventionist had over twenty years experience in community outreach, case management, education, drug treatment, and HIV counseling. He was also a trained phlebotomist and performed all biological specimen collection for HIV and hepatitis B and C antibody screening. The back-up interventionist had similar education and experience, including phlebotomy training. Both interventionists received extensive training on the two experimental interventions and standard-of-care control using an interactive skills building approach. Pilot rehearsals with 8 couples were conducted prior to subject enrollment. All HIV-CT interventions were manualized to enhance training [38, 39]. Ten percent (10%) of the interventions in each treatment condition were randomly selected for monitoring by the Project Director or Principal Investigator to assess fidelity. Fidelity assessment included a checklist and monitor notes. Adherence to protocols and intervention fidelity was discussed with interventionists at monthly project meetings. 

### 2.5. Measures

The quantitative assessment survey included measures on demographics and life history events, physical and mental health status, drug use and treatment history, sexual risk behavior, primary relationship characteristics, social network attributes and peer norms, and knowledge and attitudes regarding HIV risk. Biological measures included antibody screening for HIV-1, HBV, and HCV and urinalysis for consumption of marijuana, cocaine, opioids, and amphetamines. The current analysis focuses on the effects of treatment condition on a composite measure of HIV risk (primary outcome) controlling for selected covariates. 

#### 2.5.1. Dependent (Outcome) Variables

Analyses examining separate HIV risk behavior outcomes, such as percent condom use, can yield misleading results in HIV prevention trials because participants might reduce their risk in one risk behavior or set of behaviors while increasing risk in another set of behaviors, a phenomenon known as risk compensation. For example, participants might compensate for increasing condom use by concomitantly increasing frequency of intercourse [40]. To overcome this potential limitation we constructed a priori a composite measure of overall HIV risk as the primary outcome. The composite measure is an estimate of the probability of an uninfected individual acquiring HIV infection over a given time period (1 year) based on multiple self-reported drug and sexual risk behaviors, weighted by the risk of transmission (infectivity) associated with each risk behavior and the HIV status of various risk partners. This HIV composite risk score (probability of becoming infected) is calculated separately for each individual in the sample. One advantage of using this type of outcome measure is that HIV incidence rates for each treatment group can be estimated from the individual HIV risk probabilities [41, 42]. An estimate of relative risk can then be computed comparing treatment groups by the number of HIV infections prevented per 1000 person years. This outcome metric is analogous to HIV seroconversion end points used in larger prevention trials and is intuitively interpretable: intervention A prevented *x* more HIV infections compared to intervention B over a given time period. A further advantage is that estimated HIV incidence can be decomposed to reveal the contribution of each specific risk behavior to overall HIV risk and risk reduction. The composite HIV risk measure was calculated using a Bernoulli-process model first introduced by Pinkerton et al. and Holtgrave et al. [41, 42]. The HIV risk behaviors included in the composite measure and the risk parameters used to weight the model, as well as the Bernoulli formula, are presented in Table 1. Risk parameter values were culled from recently published data.

As a supplement to analysis using the composite HIV risk outcome, we modeled the effects of treatment on a set of secondary outcomes consisting of specific risk behaviors. These supplemental analyses were performed to foster comparability with previous studies and explore components of overall risk reduction.

#### 2.5.2. Independent Variable

*Treatment Condition.* The primary independent variable of interest consisted of the three treatment (intervention) conditions: couple-based HIV-CT, woman-only relationship-focused HIV-CT, and NIDA standard HIV-CT (control).

#### 2.5.3. Covariates

*Demographics*. Study participants reported on their age, race/ethnicity, employment status, educational level, and residence type. 

*Risk Profile*. Data were also collected on whether respondents were ever diagnosed with a sexually transmitted disease, ever traded sex for drugs or money, and ever injected illicit drugs. 

*Relationship Characteristics. *Couple attributes included marital status, relationship duration (years), and whether or not the couple was currently trying to conceive. Perceived relationship closeness, often related to couples' risk behavior, was measured using the Inclusion of Other in Self (IOS) interpersonal closeness scale dichotomized at mid point [47]. 

*Risk Behavior*. Couples' risk measures (over prior 3 months) included use of injection drugs and number and percent of receptive syringe sharing events and number and percent of unprotected and condom-protected vaginal and anal sex acts. Risk with secondary (i.e., nonprimary) sex partners included condom use and number of secondary partners, as well as receptive syringe sharing. Couples' HIV serodiscordant status was based on male and female self-report (all females tested HIV negative).

### 2.6. Sample Size Determination

Power calculations were performed using SAS IML (ver. 8.2) to determine the appropriate sample size prior to conducting the study (with 0.05 alpha and 0.80 power). Means and variances for outcome measures, and other model parameters, were obtained empirically from data previously collected from the study population. An effect size estimate of Cohen's *d* = 0.28 (or about 1.5 HIV infections averted in our primary outcome metric) was derived from a meta-analytic review of HIV counseling and testing efficacy studies, which reported mean effect sizes for intervention-related effects for couples and individuals [48]. Power calculations based on an intention-to-treat (ITT) multi-level analytic model indicated a sample size of 315 enrolled couples to detect this magnitude of effect. A total of 330 couples were enrolled in the study to ensure adequate power. 

### 2.7. Statistical Analysis

To assess whether randomization achieved comparability on baseline characteristics across treatment conditions, we performed Welch's ANOVA for continuous variables and two-tailed Fisher's Exact tests using 2 (dichotomous) × 3 (treatment conditions) contingency tables for dichotomous variables. Fisher's exact tests were also performed separately on data from female and male participants at each followup point to assess differential attrition across treatment conditions. We further examined the effects of baseline characteristics on attrition and on treatment-by-attrition interactions to test for differences between participants lost and retained to followup and differential loss across intervention conditions. Bonferroni adjustment to *P* values was applied to attrition analysis due to the large number of hypothesis tests performed. 

To test the primary study hypothesis regarding intervention effects on women's composite HIV risk, we conducted repeated measures generalized linear mixed model (GLMM) analysis [49] with robust model estimation techniques using SAS PROC GLIMMIX (ver. 9.2). The use of GLMM facilitates adherence to the ITT approach in that observations from all randomized participants are included. The dependent variable in the model was composite HIV risk (described previously); the independent variable was treatment condition (3 arms); baseline composite HIV risk was included as a covariate; baseline variables with *P* values ≤ 0.15 when regressed on treatment condition and variables that were associated with attrition rate were also added as covariates [50]. Finally, followup assessment time (3 or 9 months after baseline) and time-by-treatment interaction effects were specified in the model. Robust estimation methods—restricted maximum likelihood estimation, Newton-Raphson optimization, radial smoother model fit, and a residual-based sandwich estimator for covariance matrix parameter estimates—were used to produce stable estimates in the presence of potential residual outliers. Noncensored missing data (of which there was <4%) were handed with the EM likelihood function under the assumption of MCAR. Model parameters were weight-adjusted in accordance with standard procedures to adjust for sample network structure due to RDS recruitment [51]. Due to the distributional properties of the outcome variable, the response was modeled as a Poisson distribution with a log link function. 

Sensitivity analysis was performed to explore the sensitivity of study results to changes in the estimated HIV risk parameters used to model composite HIV risk scores. We examined the effects of doubling (high) or halving (low) model base estimates for each parameter on intervention efficacy (HIV infections averted relative to controls). We also determined the effects on study results of doubling or halving all parameters in the model simultaneously.

As a supplemental analysis, we further examined the effects of treatment on individual risk behavior outcomes (e.g., percent condom use). Fisher's exact test was used to assess differences in proportions between treatment conditions on individual risk behaviors at 9-month followup. For risk behaviors reported as count data, *t* statistics were estimated by zero-inflated negative binomial regression [52] using SAS PROC COUNTREG (ver. 9.2).

## 3. Results 

### 3.1. Participant Flow and Retention

**Figure 1:**
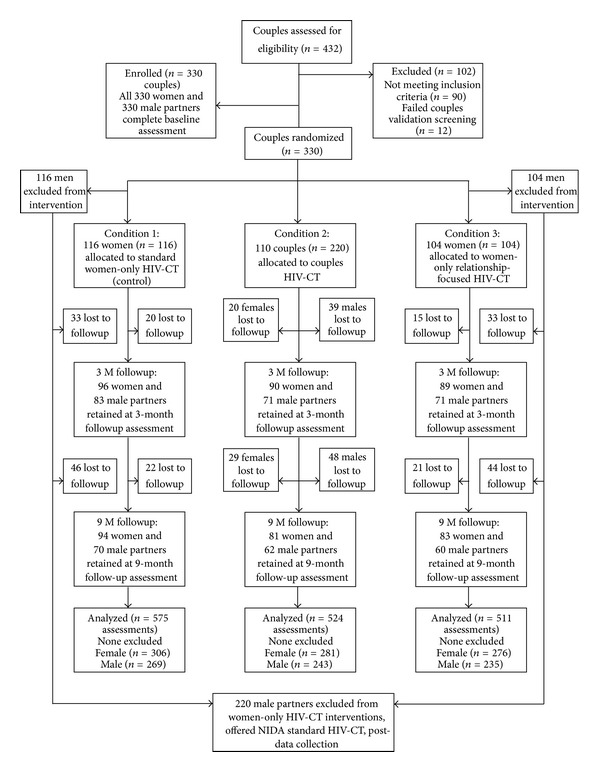
Flow diagram of participant progress through the phases of the randomized trial.

A participant flow diagram is presented in Figure 1. A total of 432 women were screened for eligibility, of which 102 were deemed ineligible, mainly due to lack of a primary partner or drug use criteria. The remaining 330 women and their primary male partners were enrolled in the study and randomized to one of the three treatment arms. Retention rates were 83% for women and 69% for men at 3 month followup and 79% for women and 58% for men at 9-month followup. The average number of days between baseline and 3 month followup was 94 (Std. Dev., 13.2; *n* = 275); and between 3 month and 9 month followup was 182 days (Std. Dev., 18.5; *n* = 259). Retention rates did not differ significantly by the treatment group for either women or men (Table 2). There were no significant differences among treatment conditions on baseline characteristics indicating that randomization achieved balanced groups (Table 3). Several significant differences were observed on baseline characteristics between participants lost to followup and those retained (Table 4). African American race/ethnicity predicted a higher retention rate compared to Hispanic race/ethnicity (83.6% versus 69.9%, *P* < 0.001) at 3-month followup; mean age of those lost to followup (*M* = 38.2) was slightly younger than those retained (*M* = 39.8), at both 3-month (*F*[2,517] = 4.31, *P* = 0.04) and 9-month (*F*[2,517] = 5.94, *P* = 0.02) followup; and couples lost to followup had been together fewer years compared to couples retained, at 3-month (*M* = 6.0 versus 8.1, *F*[2,517] = 12.0, *P* ≤ 0.001) and 9-month (*M* = 5.9 versus 8.4, *F*[2,517] = 20.6, *P* ≤ 0.0001) assessment. No treatment-by-attrition interactions on baseline characteristics were observed.

### 3.2. Treatment Effects on Women's HIV Risk: Primary Outcome

**Figure 2:**
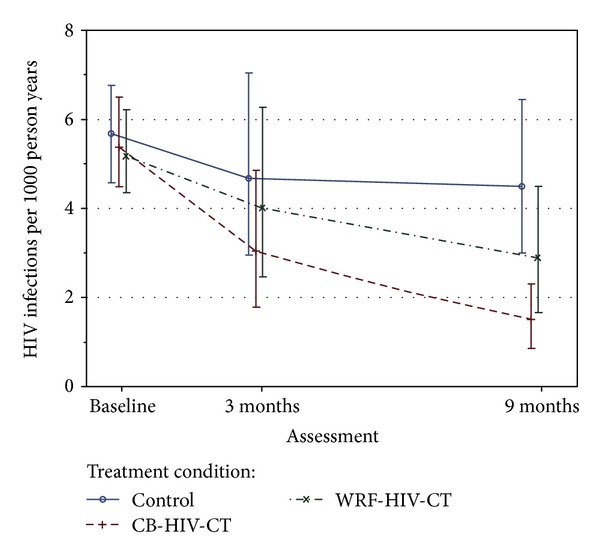
Composite HIV risk and 95% confidence intervals by treatment group and followup assessment.

Results of the repeated measures GLMM analysis, which tested the effects of the two experimental HIV-CT interventions against the NIDA standard control, are depicted in Figure 2. The graph displays the least squares means and 95% confidence intervals on composite HIV risk for 330 female HIV seronegative subjects by treatment group and assessment point, adjusting for HIV risk at baseline, race/ethnicity, age, employment status, STD history, and relationship duration. As expected, treatment group means on women's composite HIV risk were closely clustered at preintervention (baseline) assessment indicating that randomization achieved comparability on the primary outcome. The group-by-time interaction effect, which tests for significant group differences in the rate of change in HIV risk over the 9 month assessment period, indicated a significant decrease in HIV risk in the couple-based HIV-CT intervention group compared to that of the control group (*b* = −0.51, *t*(527) = −3.20, *P* = 0.0015) and compared to that of the women's relationship-focused-HIV-CT group (*b* = −0.34, *t*(527) = −2.07, *P* = 0.039), but no significant difference between WRF-HIV-CT and controls (*b* = −0.17, *t*(527) = −1.09, *P* = 0.28).

At 3-month postintervention assessment, the WRF-HIV-CT averted 0.59 more HIV infections per 1000 py than the control condition, but this estimated sample effect did not reach statistical significance (*P* = 0.67). HIV risk reduction in the CB-HIV-CT group compared to controls at 3 months also did not attain statistical significance (1.61 HIV infections averted per 1000 py, *P* = 0.21). At 9-month followup assessment, observed reduction in HIV risk in the WRF-HIV-CT group relative to the control group was not statistically significant (1.65 HIV infections averted per 1000 py, *P* = 0.14). Women's HIV risk at 9-month assessment was significantly reduced in the CB-HIV-CT group compared to both the NIDA standard HIV-CT control group (3.04 HIV infections averted per 1000 py, *P* = 0.0004) and the WRF-HIV-CT group (1.39 HIV infections averted per 1000 py, *P* = 0.05). These ITT-based inferential findings did not change when analyses were performed on the subset of completer cases (*n* = 234). 

Overall model-estimated composite HIV risk for women in the couple-based HIV-CT group at 9-month followup was 1.47 HIV infections per 1000 py (95% CI: 0.91, 2.36) compared to 4.51 HIV infections per 1000 py (95% CI: 3.09, 6.56) for women in the control group. Thus, at terminal followup the CB-HIV-CT intervention averted 3.04 more HIV infections per 1000 py than the control intervention (*P* = 0.0004). Prepost comparison also indicates a significant reduction of overall HIV risk in the CB-HIV-CT group from baseline (5.44 HIV infections per 1000 py) to 3-month assessment (3.02 HIV infections averted per 1000 py, *P* = 0.02) and from 3-month to 9-month assessment (1.47 HIV infections averted per 1000 py, *P* = 0.02). 

The relative contribution of specific HIV risk factors toward overall risk reduction can be estimated by decomposition of the Bernoulli model into subcomponents. Of the 3.04 HIV infections averted per 1000 py at 9-month followup in the CB-HIV-CT group compared to the control group, 0.95 infections (31%) were averted through reductions in vaginal sex risk within primary couples; 0.77 HIV infections (25%) were averted by reductions in injection risk behavior within primary couples; 0.74 infections (24%) were averted by reductions in anal sex risk within primary couples; 0.48 infections (16%) were averted by women's reduction in sexual risk with secondary sex partners; and 0.10 HIV infections (3%) were averted by women reducing their injection risk with drug-using partners who were not their primary sex partner. 

### 3.3. Sensitivity Analysis

Modifications to risk parameter estimates of the HIV composite risk model did not change the study findings. All analyses assessing intervention group differences that produced nonsignificant results (*P* > 0.05) with base model estimates also produced nonsignificant results using either high or low model estimates, including simultaneous high or low estimates. Likewise, all significant results using base estimates remained statistically significant (*P* < 0.05) in the sensitivity analysis. Effect sizes were influenced most by modifications to *π*_1_ (probability that a primary partner is HIV infected, when HIV status is unknown). For example, at the 9-month assessment, when *π*_1_ was doubled the CB-HIV-CT intervention averted 4.19 HIV infections per 1000 py relative to controls (*P* < 0.001), compared with 3.04 infections averted with base estimates, and when *π*_1_ was halved the CB-HIV-CT intervention averted 2.13 infections per 1000 py compared to controls (*P* < 0.001). For the same comparison, when all risk parameters in the model were simultaneously doubled (higher risk) or simultaneously halved (lower risk), the CB-HIV-CT intervention averted 5.28 (*P* < 0.001) and 1.43 (*P* < 0.001) infections per 1000 py more than controls, respectively.

### 3.4. Secondary Outcomes

To further explore the components of overall HIV risk reduction observed in the couple-based HIV-CT group compared to the control group at 9-month followup, we examined differences in individual risk behaviors by treatment (Table 5). Among women who reported injecting with their primary male partner, those in the CB-HIV-CT group compared to controls reported significantly less frequent receptive syringe sharing with primary partners (*M* = 1.4 versus 8.3, *P* = 0.0002). This reduction accounted for 25% of the overall HIV risk reduction in the CB-HIV-CT group relative to the control group. In addition, a trend was observed toward less frequent receptive syringe sharing with persons other than a primary sex partner among those in the CB-HIV-CT group (0.1 versus 1.4, *P* = 0.08). Frequency of unprotected anal intercourse with a primary male partner was also lower in the CB-HIV-CT group compared to that of controls (*M* = 0.7 versus 5.7, *P* = 0.005). Whereas observed reductions in vaginal sex risk among couples (e.g., number of unprotected acts of vaginal intercourse) in the CB-HIV-CT relative to controls did not reach statistical significance (*M* = 15.4 versus 22.2, *P* = 0.13), the weighted HIV transmission model based on vaginal sex behaviors indicated a reduction of 0.95 HIV infections per 1000 py in the CB-HIV-CT group. 

## 4. Discussion

Couple-Based HIV counseling and testing demonstrated significant reductions in women's overall HIV risk compared to both the NIDA standard HIV-CT (control) and woman-only relationship-focused HIV-CT at 9-month postintervention. There were no statistically significant reductions in overall HIV risk in the woman-only relationship-focused HIV-CT compared to the NIDA control. The couple-based intervention, designed for women substance users and their primary male partners, showed reduced HIV risk from baseline to 3- and 9-month assessments. The evidence indicates that a brief HIV counseling and testing risk-reduction intervention administered jointly to both male and female members of drug-using couples is more effective in reducing HIV risk among women with primary partners than counseling and testing interventions administered exclusively to women. The couple-based HIV-CT intervention was most effective at reducing injection risk among primary partners. Vaginal and anal sex risk with primary partners and sexual risk with secondary partners also contributed to a significant reduction of overall HIV risk among couple-based intervention participants compared to controls at 9-month assessment. Reductions in injection risk with secondary partners contributed substantially less to overall risk reduction. 

The HIV counseling and testing mode of intervention delivery, although brief, has several important advantages over interventions involving multiple sessions, particularly for illicit drug users. HIV-CT is an existing health service that is already accessible and utilized by members of communities characterized by high HIV incidence and prevalence, such as Harlem and South Bronx in New York City. Moreover, the often chaotic and transient lives of drug users (especial out-of-treatment drug users) often preclude attendance at multiple intervention sessions over many weeks or months. By contrast, HIV-CT consists of a single pretest and testing session followed by a single posttest session. Participation is often initiated by clients seeking testing who are motivated to learn their HIV status. CDC guidelines recommend recurrent HIV counseling and testing at least annually for persons in high-risk categories [53]. Thus, evidence of HIV risk reduction over a 9-month period may indicate acceptable sustainability of the couple-based HIV-CT, assuming annual repeated testing. Typically, HIV prevention interventions reach peak risk reduction several weeks or months postintervention then begin to decay [54]. Examining the trends in the sample point estimates over time, this is evident for the NIDA standard control group, which showed little or no additional reduction of risk after the 3-month assessment. By contrast, HIV risk continued to decline in the couple-based HIV-CT group beyond the 3-month assessment point, although the rate (slope) of reduction decreased after 3 months (see Figure 2). This might be due to the reactive effects of the 3-month assessment, which may have served as a “booster” to the intervention. An alternative explanation is that the couple-based intervention initiated changes in behavior among sexual dyads that resulted in a pattern of sustained risk reduction, for example, by continued improvement of communication skills. 

In a recent systematic review of the literature, Burton et al. [55] identified six studies that used rigorous comparative designs to assess the efficacy of couple-based behavioral interventions for HIV prevention. Although heterogeneity of study characteristics precluded a formal meta-analysis, the authors concluded that couple-based HIV prevention interventions consistently reduced sexual risk behavior compared to control interventions. Two of the six couple-based HIV prevention studies identified by Burton et al. employed an HIV counseling and testing delivery model. In a multi-site randomized trial conducted in Kenya, Tanzania, and Trinidad, Coates et al. [56] found that men assigned to couple-based HIV-counseling and testing reported reduced unprotected intercourse with both primary and nonprimary partners compared with controls. A prospective cohort study in Kenya found that women who received HIV-CT together with their primary male partner reported marginal increases in condom use compared to women receiving individual HIV-CT [57]. 

Only two studies identified by Burton et al. employed randomized controlled trial designs and were conducted in the United States. El-Bassel et al. [58, 59] randomly assigned women recruited from hospital clinics in Bronx, NY, to one of three treatment arms: a couples joint intervention, an individual (woman-only) relationship-focused intervention, or an individual (woman-only) educational control. No differences were detected in risk behavior between the couple-based and woman-only relationship-focused interventions, but both of these interventions were found to be effective at reducing unprotected intercourse compared to the educational control, at both 3- and 12-month followup assessments. The two experimental conditions each consisted of six weekly 2-hour sessions, and the drop-out rate prior to and after the first orientation session was high, indicating the need for brief intervention delivery modalities. In another RCT conducted in Los Angeles, Harvey et al. [60] randomized Hispanic heterosexual couples to either a single-session couples-focused HIV/AIDS risk reduction intervention or a couple-based educational control (both interventions were delivered to groups). There were no observed differences between the couples risk reduction intervention and control condition on self-reported condom use. However, the study may have had limited power to detect a difference between treatment conditions on a single risk factor such as condom use. This indicates the need for larger sample sizes and measures of HIV risk that incorporate multiple risk behaviors. 

The Harlem River Couples Project is unique in that it combines (1) a randomized clinical trial design to evaluate a brief couple-based HIV counseling and testing intervention for women and their primary male partners conducted in the USA; (2) both drug-related and sexual risk reduction components within the primary relationship as well as outside the primary relationship in the interventions and assessment; and (3) a primary outcome measure based on composite HIV risk modeled on transmission probabilities using an epidemiological metric—the estimated number of HIV infections averted per 1000 person years (py). 

The current study also has several limitations. Use of an adaptive sampling approach that combined targeted sampling with respondent driven sampling is unlikely to have produced a truly representative sample of drug-involved heterosexual couples. A recent comparison of these two strategies indicate that RDS tends to recruit more racial/ethnic minorities, older individuals, and the homeless and is best suited for studies involving both IDUs and nonIDUs, as well as those examining both injection and sexual risk behaviors. In contrast, targeted street outreach captures more injection drug users and is more appropriate for recruiting samples of high-risk IDUs [61]. The demographic and risk behavior characteristics of our drug-using sample (mostly minority, out-of-treatment, high sexual risk behaviors, one-third IDU) reflect the inherent tendencies of both recruitment strategies, but also are consistent with the population for which the experimental interventions were designed, with the exception of older age. The mean age of women enrolled in the study was 38.4 years, and only 10% of the sample was younger than 25 years. The older age of the sample is typical for HIV prevention studies involving drug users. A meta-analysis of 33 US-based HIV intervention trials with drug users found a mean age of 36 years [62]. Targeted sampling and RDS, methods commonly used to recruit drug users in HIV prevention studies, have been shown to produce older samples [61, 63, 64]. Volunteerism and masking—over or under recruitment of participants based on willingness—might also have favored older participants due to differential acceptability, access, and cost/benefit assessment. Given the under representation of younger women in the trial, it is unclear whether the observed intervention effects will generalize to young women substance users with primary male partners. One potential source of masking bias is related to the exclusion of women who self-reported feeling uncomfortable or threatened participating in a couple-based HIV prevention intervention. This exclusion criterion was implemented for ethical reasons but also because the intervention was not designed to address the needs of more volatile and violent relationships (more common among younger couples), and our study findings do not generalize to couples in these types of relationships. Analyses indicate that participants of Hispanic ethnicity, younger age, and those in newer relationships were more likely to be lost to followup, thus introducing potential bias and further potential limits on generalizability. However, these differential attrition rates were consistent across treatment conditions. Nonetheless, our relatively high attrition rate—22% of couples dropped out by terminal followup—is another potential source of selection bias if dropouts were related to unobserved variables. Another limitation is the potential lack of reliability of self-reported data collected in this trial. A-CASI data collection techniques and other interview methods were used to maximize reliability of self-reported data.

## 5. Conclusions

Sexual and injection-related transmission of HIV to women substance users often occurs in the context of risk with a primary male partner. Previous individual-based HIV risk reduction interventions have not fully leveraged the essential roles played by both members of interacting sexual dyads and attendant relationship factors in sexual decision making and risk behavior. Couple-based HIV prevention interventions targeting risk behaviors among urban women and couples in the USA have typically involved multiple sessions and high drop-out rates or single-session group interventions showing small effects. Previous studies have demonstrated the efficacy of brief couple-based HIV counseling and testing interventions for sexual risk reduction but thus far only in Africa. The current randomized trial demonstrates the efficacy of couple-based HIV counseling and testing designed to address both drug-related and sexual risk behaviors among substance using heterosexual couples in the United States in an urban setting.

## Figures and Tables

**Table 1:** Bernoulli formula, estimated risk parameters, and self-reported behaviors used to create composite HIV risk outcome measure (HIVRISK).

Bernoulli mathematical formula		

HIVRISK = 1 − (1 − A)(1 − B)(1 − C)		
A = *π*_1_[1 − (1−*α*_*v*_)^mvu^(1−*εα*_*v*_)^mvp^(1−*α*_*a*_)^mau^(1−*εα*_*a*_)^map^(1−*α*_*j*_)^mij^]		
B = *π*_2_[1 − [(1−*α*_*v*_)^svu^(1−*εα*_*v*_)^svp^]^sv^[(1−*α*_*a*_)^sau^(1−*εα*_*a*_)^sap^]^sa^]		
C = *π*_3_[1 − [(1−*α*_*j*_)^sij^]^sj^]		

Estimated HIV risk parameters	Base estimates	Source

*π*_1_: probability that primary male sex partner is HIV infected	1.0 | .15 | .05	^†^
*π*_2_: probability that a secondary sex partner is HIV infected	0.15	^‡^
*π*_3_: probability that an injection partner is HIV infected	0.25	^‡^
α_a_: per contact probability of HIV transmission (infectivity) for anal sex	.017	[43, 44]
α_v_: per contact HIV transmission for male-to-female vaginal sex	.0008	[43]
α_j_: per act HIV transmission for receptive syringe sharing	.0074	[45, 46]
ε: condom failure rate	.10	[44]

Measured self-reported risk behaviors		

mvu: number of acts of condom unprotected vaginal intercourse with primary male partner		
mvp: number of acts of condom protected vaginal intercourse with primary male partner		
mau: number of acts of condom unprotected anal intercourse with primary male partner		
map: number of acts of condom protected anal intercourse with primary male partner		
mij: number of times receptive syringe sharing with primary male partner		
svu: number of acts of condom unprotected vaginal intercourse with secondary sex partner		
svp: number of acts of condom protected vaginal intercourse with secondary sex partner		
sau: number of acts of condom unprotected anal intercourse with secondary sex partner		
sap: number of acts of condom protected anal intercourse with secondary sex partner		
sij: number of times receptive syringe sharing with secondary partner		
sv: number of secondary vaginal sex partners		
sa: number of secondary anal sex partners		
sj: number of secondary injection partners		

A represents HIV risk from a primary male partner; B is HIV risk from a secondary sex partner; C is HIV risk from a secondary injection partner.

^†^Based on self-reported HIV status of male partner: self-reported HIV positive = 1.0; self-reported HIV negative/unaware and IDU = 0.15; self-reported HIV negative/unaware and non-IDU = .05.

^‡^Based on unpublished data on HIV prevalence among IDU and non-IDU men in Harlem and South Bronx, NY, USA.

**Table 2:** Retention rates at 3- and 9-month follow-up assessments by treatment condition.

	Couples-based		Women-only relationship-focused		Control: NIDA standard		
	HIV-CT (*n* = 110)		HIV-CT (*n* = 104)		HIV-CT (*n* = 116)		*P* value
	*n *	%	*n *	%	*n *	%	
Female (*n* = 330) retention rate							
3-month followup	90	81.8	89	85.6	96	82.8	.74
9-month followup	81	73.6	83	79.8	94	81.0	.37
Male (*n* = 330) retention rate							
3-month followup	71	64.5	71	68.3	83	71.6	.53
9-month followup	62	56.4	60	57.7	70	60.3	.82

**Table 3:** Baseline characteristics of participants by treatment condition.

	Couples-based		Women-only relationship-		Control: NIDA standard			Fisher's exact test
	HIV-CT (*n* = 110)		focused HIV-CT (*n* = 104)		HIV-CT (*n* = 116)		*F* value	
	*n*	%	*n*	%	*n*	%		*P* value
Female characteristics								
Race/ethnicity								.08
African American	45	40.9	32	30.8	35	30.2		
Hispanic	56	50.9	51	49.0	64	55.2		
White/other	9	8.2	21	20.2	17	14.6		
Unemployed	88	80.0	89	85.6	87	75.0		.15
Completed high school	52	47.3	52	50.0	54	46.6		.89
Homeless^a^	14/109	12.8	17/104	16.4	16/116	13.8		.77
Ever diagnosed with STI^b^	60/103	58.3	45/98	45.9	65/113	57.5		.15
Traded sex last 3 months	28	25.5	21	20.2	29	25.0		.61
Ever injected illicit drugs	47	42.7	53	51.0	57	50.9		.39
Age (yrs)^†^		38.83 (8.6)		37.63 (8.3)		38.67 (8.3)	0.65^‡^	.52^‡^
HIV transmission risk^†^		0.96 (1.76)		0.99 (1.73)		0.86 (1.45)	0.21^‡^	.81^‡^

Couple characteristics								
Marital status								.32
Married, legal	25	22.7	27	26.0	33	28.5		
Married, common law	73	66.4	64	61.5	77	66.4		
Not married	12	10.9	13	12.5	6	5.2		
Trying to conceive	21	19.1	20	19.2	16	13.8		.48
Perceived closeness	77	70.0	77	74.0	83	71.6		.81
Condom use last 3 months	26	23.6	25	24.0	22	19.0		.59
HIV serodiscordant	8	7.3	8	7.7	6	5.2		.73
Prob (%) male HIV positive^†a^	109	16.2	104	17.6	116	14.9	0.39^‡^	.68
Relationship duration (yrs)^†c^	108	7.0 (7.5)	104	7.5 (7.5)	116	8.3 (7.3)	0.86^‡^	.42

^†^Mean and standard deviation given.

^‡^Welch's ANOVA *F* statistic and *P* value.

^a^Variable has 1 missing response.

^b^Variable has 16 missing responses.

^c^Variable has 2 missing responses.

**Table 4:** Baseline characteristics of participants lost to follow-up compared to those retained.

	3-month followup (*n* = 660)			9-month followup (*n* = 660)		
	Lost (*n* = 160)	Retained (*n* = 500)	*P* value	Lost (*n* = 210)	Retained (*n* = 450)	*P* value
	%	%		%	%	
Participant characteristics						
Race/ethnicity			**<.001**			.10
African American	23.8	38.8		30.0	37.6	
Hispanic	67.5	50.2		60.5	51.6	
White/other	8.8	11.0		9.5	10.9	
Unemployed	76.9	72.0	.26	73.3	73.1	1.00
Completed high school	49.4	52.4	.53	51.0	52.0	.80
Homeless^a^	27.0	29.8	.55	30.0	28.7	.78
Ever diagnosed with STI^b^	40.7	46.0	.26	47.1	43.6	.43
Ever being injected with illicit drugs	56.3	50.0	.17	53.3	50.7	.56
Age (yrs)^†^	38.2	39.8	**4.31, .04**	38.2	39.9	**5.94, .02**
HIV transmission risk^†^	2.03	1.61	0.57, .45	1.31	1.78	1.47, .22

Couple characteristics						
Marital Status			.07			.37
Married, legal	18.1	26.6		23.8	24.9	
Married, common law	70.6	65.0		64.8	67.1	
Not married	11.3	8.4		11.4	8.0	
Trying to conceive	10.6	8.0	.33	7.6	9.1	.56
Perceived closeness	75.0	75.2	1.00	73.8	75.8	.63
Condom use last 3 months	21.9	20.8	.82	23.8	19.8	.26
Anal sex last 3 months	33.8	28.4	.20	31.4	28.9	.52
HIV serodiscordant (*n* = 330)	7.3	6.6	.77	5.6	7.0	.79
Prob (%) male HIV positive^†^	.18	.16	0.22, .64	.14	.17	0.58, .45
Relationship duration (yrs)^†^	6.0	8.1	**12.0**, **<.001**	5.9	8.4	**20.6**, **<.0001**

^†^Mean given.

^‡^Welch's ANOVA *F* statistic.

**Table 5:** Comparison of proportions and means between couples-based HIV-CT and control conditions on selected risk behaviors at 9-month followup. *T* statistic for differences in count data by treatment based on zero-inflated negative binomial regression.

Prior to 3-month self-reported HIV risk behavior at 9-month followup assessment	Couples HIV-CT (*n*)	Control HIV-CT (*n*)	Estimate (SE)	*t* statistic	*P* value
Injection risk with primary partner^a^					
% receptive syringe sharing^b^	12.4 (18)	11.7 (13)			.90
# times receptive syringe sharing^c^	1.4 (18)	8.3 (13)	−1.47 (0.40)	−3.73	.0002
Vaginal sex risk with primary partner					
% unprotected vaginal sex	75.0 (81)	82.6 (94)			.72
% condom use (vaginal sex)	23.5 (81)	21.3 (94)			.86
# unprotected vaginal sex acts	15.4 (81)	22.2 (94)	−0.37 (0.24)	−1.51	.13
Anal sex risk with primary partner					
% unprotected anal sex	19.8 (81)	14.9 (94)			.43
% condom use (anal sex)^d^	27.8 (18)	35.7 (14)			.71
# unprotected anal sex acts	0.7 (81)	5.7 (94)	−2.13 (0.75)	−2.83	.005
Sexual risk with secondary partner(s)					
% with secondary partner(s)	19.8 (81)	20.2 (94)			.99
% condom use (secondary partner)^e^	79.0 (16)	68.8 (19)			.70
# of secondary partners	0.8 (81)	0.4 (94)	0.70 (0.47)	1.49	.14
Injection risk with nonprimary partner					
% who injected with NP partner(s)	9.9 (81)	5.3 (94)			.27
# times receptive syringe sharing^f^	0.1 (8)	1.4 (5)	−2.45 (1.41)	−1.73	.08

^a^Subsample of injection drug users.

^b^Fisher's exact test used to assess differences in proportions between treatment conditions.

^c^Raw means shown for each treatment group. *T* statistic based on zero-inflated negative binomial regression for count data to assess group differences.

^d^Subsample of women reporting anal sex with primary partners.

^e^Subsample of women reporting at least 1 secondary sex partner.

^f^Subsample of women who injected with persons other than their primary sex partner.
